# The *Macrophage Migration Inhibitory Factor* (*MIF*) Promoter Polymorphisms (rs3063368, rs755622) Predict Acute Kidney Injury and Death after Cardiac Surgery

**DOI:** 10.3390/jcm9092936

**Published:** 2020-09-11

**Authors:** Luisa Averdunk, Jürgen Bernhagen, Karl Fehnle, Harald Surowy, Hermann-Josef Lüdecke, Sören Mucha, Patrick Meybohm, Dagmar Wieczorek, Lin Leng, Gernot Marx, David E. Leaf, Alexander Zarbock, Kai Zacharowski, on behalf of the RIPHeart Study Collaborators, Richard Bucala, Christian Stoppe

**Affiliations:** 1 Department of Intensive Care Medicine, University Hospital Aachen, Rheinisch Westphälische Technische Hochschule Aachen, 52074 Aachen, Germany; luisa.aver@gmail.com (L.A.); gmarx@ukaachen.de (G.M.); 2 Institute of Human Genetics and Department of Pediatrics, Medical Faculty, Heinrich Heine University, 40225 Düsseldorf, Germany; harald.surowy@uni-duesseldorf.de (H.S.); Hermann-Josef.Luedecke@uni-duesseldorf.de (H.-J.L.); dagmar.wieczorek@hhu.de (D.W.); 3 Department of Vascular Biology, Institute for Stroke and Dementia Research, Klinikum der Universität München, Ludwig-Maximilians-University Munich, 80333 Munich, Germany; Juergen.Bernhagen@med.uni-muenchen.de; 4 German Center for Cardiovascular Research (DZHK), Partner Site Munich Heart Alliance, 10785 Berlin, Germany; 5 Munich Cluster for Systems Neurology (EXC 2145 SyNergy), 81377 Munich, Germany; 6 Algora: Statistics and Clinical Research GmbH, 85540 Haar, Germany; karl.fehnle@algora.de; 7 Institute of Clinical Molecular Biology, Christian Albrechts University of Kiel, 24118 Kiel, Germany; s.mucha@ikmb.uni-kiel.de; 8 Institute for Cardiogenetics, University of Lübeck, Ratzeburger Allee 160, 23562 Lübeck, Germany; 9 Department of Anesthesiology, Intensive Care Medicine & Pain Therapy, University Hospital Frankfurt, Goethe University, 60323 Frankfurt, Germany; meybohm_p@ukw.de (P.M.); kai.zacharowski@kgu.de (K.Z.); 10 Department of Internal Medicine, Yale University School of Medicine, New Haven, CT 06510, USA; lin.leng@yale.edu; 11 Division of Renal Medicine, Brigham and Women’s Hospital, Boston, MA 02115, USA; deleaf@bwh.harvard.edu; 12 Harvard Medical School, Boston, MA 02115, USA; 13 Intensive Care and Pain Medicine, Department of Anesthesiology, University of Münster, 48149 Münster, Germany; zarbock@uni-muenster.de; 14 Department of Anesthesiology, Intensive Care Medicine and Pain Therapy, University Hospital Würzburg, 97080 Würzburg, Germany

**Keywords:** acute kidney injury, genetic polymorphisms, risk prediction, (cardiac) surgery, inflammatory cytokines, clinical studies

## Abstract

Background: Macrophage Migration Inhibitory Factor (MIF) is highly elevated after cardiac surgery and impacts the postoperative inflammation. The aim of this study was to analyze whether the polymorphisms CATT_5–7_ (rs5844572/rs3063368,“-794”) and G>C single-nucleotide polymorphism (rs755622,-173) in the *MIF* gene promoter are related to postoperative outcome. Methods: In 1116 patients undergoing cardiac surgery, the *MIF* gene polymorphisms were analyzed and serum MIF was measured by ELISA in 100 patients. Results: Patients with at least one extended repeat allele (CATT_7_) had a significantly higher risk of acute kidney injury (AKI) compared to others (23% vs. 13%; OR 2.01 (1.40–2.88), *p* = 0.0001). Carriers of CATT_7_ were also at higher risk of death (1.8% vs. 0.4%; OR 5.12 (0.99–33.14), *p* = 0.026). The GC genotype was associated with AKI (20% vs. GG/CC:13%, OR 1.71 (1.20–2.43), *p* = 0.003). Multivariate analyses identified CATT_7_ predictive for AKI (OR 2.13 (1.46–3.09), *p* < 0.001) and death (OR 5.58 (1.29–24.04), *p* = 0.021). CATT_7_ was associated with higher serum MIF before surgery (79.2 vs. 50.4 ng/mL, *p* = 0.008). Conclusion: The CATT_7_ allele associates with a higher risk of AKI and death after cardiac surgery, which might be related to chronically elevated serum MIF. Polymorphisms in the *MIF* gene may constitute a predisposition for postoperative complications and the assessment may improve risk stratification and therapeutic guidance.

## 1. Introduction

Conventional open-heart surgery is performed annually in more than one million patients worldwide, and the incidence of postoperative sequelae including acute organ dysfunction remains high [1,2]. A more precise identification of patients’ risk for postoperative complications is desirable both for prognostic guidance and for the application of earlier and more effective interventions. While clinical scoring systems such as the well-established EuroSCORE were primarily developed for the preoperative risk stratification of mortality in cardiac surgery patients, only limited evidence exists about its value for postoperative organ dysfunction and other complications [3]. The identification of genomic risk alleles could be especially helpful to more accurately predict outcomes and to enable personalized medicine approaches.

Oxidative stress and a systemic inflammatory response contribution to the pathogenesis of postoperative organ dysfunctions following cardiac surgery [4]. Macrophage migration inhibitory factor (MIF) is a stress-regulating cytokine that increases in the circulation after cardiac surgery [5]. Within the *MIF* gene promoter, two polymorphisms, a G>C single nucleotide polymorphism 270 bases before *MIF* transcription start (−270) (originally described as −173; HGVS nomenclature: NM_002415.2 c.−270G>C, rs755622) and a CATT tetranucleotide repeat CATT_5-7_ (rs3063368), have been associated with disease severity of multiple chronic inflammatory diseases, including osteoporosis, ankylosing spondylitis, and multiple sclerosis [6,7,8]. A higher number of CATT repeats has been reported to increase *MIF* promotor activity and to be associated with higher circulating MIF concentrations in different autoimmune and chronic inflammatory conditions [9,10,11,12]. The clinical significance of functional polymorphisms in *MIF* for postoperative outcome after cardiac surgery is unknown. In this study, we analyzed 1116 patients who underwent elective cardiac surgery with a cardiopulmonary bypass. We analyzed whether *MIF* promoter polymorphisms impact the risk of postoperative organ dysfunction and mortality in patients undergoing cardiac surgery. In a subset of patients, we also examined the correlation between *MIF* genotypes and circulating MIF levels.

## 2. Materials and Methods

### 2.1. Study Design and Patients

The present study is a predefined sub-study performed in cardiac surgery patients of the Remote Ischemic Preconditioning Heart (RIPHeart) study (January 2011–May 2014), which investigated whether upper limb remote ischemic conditioning reduced mortality and the incidence of myocardial infarction, stroke, and acute kidney injury (AKI) in adults scheduled for elective cardiac surgery requiring a cardiopulmonary bypass [13]. As the initial intervention study did not show group differences, this *MIF* polymorphism study includes all patients irrespective of the initial group assignment [13]. The trial was undertaken in compliance with International Conference on Harmonisation Good Clinical Practice guidelines, the Declaration of Helsinki (2008), and European Directive 2001/20/CE regarding the conduct of clinical trials (4 April 2001). The study was registered at ClinicalTrials.gov (NCT01067703). The study protocol was approved by the ethics committees of the University of Kiel, Aachen, and all participating centers of this prospective multicenter study.

Patients scheduled for elective cardiac surgery with use of cardiopulmonary bypass (e.g., coronary artery bypass graft (CABG), valve surgery, ascending aorta replacement) were eligible for this study. Of the 1403 patients screened for the study, blood samples were available from 1196 patients. Seven patients were excluded due to missing outcome data and 70 patients were excluded due to missing genotype information (Supplemental Figure S1). Thus, data from 1119 patients were included in the current study. The CATT_8_ genotype was excluded from analysis because of its low frequency (*N* = 3), which is in accord with prior reports [14].

Blood samples were collected before surgery, at 45 min after cardiopulmonary bypass (CPB) initiation, at 2 min after opening of the cross-clamp (reperfusion), at 15 min after weaning from CPB, and at 1, 6, and 24 h after admission to ICU. Blood samples were processed no later than 2 h after collection and were stored at −70 °C or −20 °C until further transfer. The final study visit took place either before hospital discharge, or at the latest 30 days after ICU admission.

### 2.2. Outcome Measures

The primary exposure was the *MIF* genotype, which we analyzed according to the following groups: alleles (e.g., carriers of at least one C allele), genotypes (e.g., GC), and individual genotype combinations (e.g., CATT_5,7_-GC). The endpoints were the association between the *MIF* genotype and the incidence of postoperative organ dysfunctions, including AKI, myocardial infarction, new onset of atrial fibrillation, stroke, delirium, and death. Each of these outcome parameters were analyzed as single events, and in the composite outcome “multiple organ dysfunction”, when patients suffered from more than one organ dysfunction.

According to the KDIGO Clinical Practice Guideline 2012, AKI was defined as a ≥ 5-fold increase of serum creatinine from baseline, and a urine output of ≤0.5 mL/kg/h for more than 6 h, or the use of renal replacement therapy within 72 h [15]. However, a total creatinine increase of ≥ 0.3 mg/dL was not considered a diagnostic criterion for AKI, as this criterion had not yet been established in 2011 when the data collection started. Non-fatal myocardial infarction was defined by biomarker values more than five times higher than the 99th percentile of the normal reference range combined with new pathological Q-waves or new left bundle branch block within the first 72 h, standard clinical criteria for myocardial infarction from 72 h on, new ischemic finding by echocardiography or angiography, or myocardial infarction diagnosed at autopsy [16]. New onset of atrial fibrillation was defined as a new onset within the first four days after surgery [13]. The occurrence of postoperative delirium was assessed with the CAM-ICU score (preoperative, 24, 48, 72, and 96 h after surgery) [17]. Stroke was defined by any new, temporary or permanent, focal or global neurological deficit, or evidence of stroke on autopsy, and was evaluated according to the National Institutes of Health Stroke Scale (≥4 points) [18].

Myocardial infarction, atrial fibrillation, and stroke were analyzed until hospital discharge with a maximum of 14 days after surgery.

### 2.3. ELISA

Serum was available from 100 patients in the RIPheart Study. Serum MIF levels were quantified by ELISA in duplicates as previously described and according to the manufacturer’s instructions (R&D Systems, Minneapolis, MN, USA) ([5]). Samples were diluted 1:10 before analysis to obtain measures in the valid assay range.

### 2.4. Nomenclature and Genotyping of the Tetranucleotide Repeat Polymorphism CATT_n_ (rs3063368)

In adherence to the U.S. National Library of Medicine dbSNP database (ncbi.nlm.nih.gov/snp/), the tetranucleotide repeat polymorphism formerly described as rs5844572, referring to the CATT_6_ allele, will be referred to with the reference SNP number rs3063368, which comprises the multiallelic repeat polymorphism CATT_n_ present at this site. The tetranucleotide repeat polymorphism is located at position chr22:23893566-23893569: (GRCh38.p12) (ncbi.nlm.nih.gov/snp/rs3063368), and based on older transcript annotations (NM_002415.2) -794 nucleotides, or based on more accurate transcript annotations (NM_002415.2) -909 nucleotides upstream of the start codon. The repeat polymorphism is a deletion, or respectively a duplication, of TTCA tetranucleotide repeats. In parallel with former publications, in this study this SNP will be referred to as a tetranucleotide 5-, 6-, 7-, or 8- fold repeat of CATT. The DNA sequence of this SNP is illustrated in Supplemental Figure S2 (UCSC Genome Browser, genome.ucsc.edu).

EDTA-anticoagulated whole blood was used for genotyping. DNA was extracted with the Autopure LS automated system according to the manufacturer’s recommendations (Qiagen, Hilden, Germany).

For the analysis of the tetranucleotide repeat (rs3063368), as formerly described, a fragment length polymorphism PCR with fluorescently labeled primers and fragment length analysis via capillary electrophoresis was applied [10]. Of note, with this technique a phase analysis with the rs755622 single nucleotide polymorphism (SNP) is not possible. DNA was amplified in a Mastercycler gradient (Eppendorf AG, Germany). The PCR reaction mix contained 2.5 µL 10× PCR buffer, 3 µL (25 mM) MgCl2, 2 µL dNTP Mix, 0.8 µL of each primer, 0.15 µL (1 U) Taq polymerase, and 13.75 μL of purified DNA, in a total end volume of 25 μL. The PCR consisted of the following steps: an initial denaturation step (95 °C, 12 min), 35 amplification cycle (95 °C, 30 s; X (primer annealing temperature see Supplemental Table S1, 30 s; 72 °C, 30 s) and a final elongation step (72 °C, 10 min). The reverse primer was labeled with 6-Carboxyfluorescein (6-FAM) [19]. The PCR was performed in a SimpliAmp Thermal Cycler (Life Technologies, Carlsbad, CA, USA). The PCR products were subjected to capillary electrophoresis on an ABI 3730XL Genetic Analyzer (Applied Biosystems, Foster City, CA, USA). Data collection was performed with Data Collection v3.0 software (Applied Biosystems, Foster City, CA, USA) and the results were analyzed by GeneMapper ID v5 software (Applied Biosystems, Foster City, CA, USA).

### 2.5. Nomenclature and Genotyping of the SNP G>C Substitution (rs755622)

The SNP rs755622 is a substitution of G>C in the non-coding region at position chr22:23,894,205 (GRCh38.p12). Based on the older transcript annotations (NM_002415.1), this SNP has been formerly described with the position NM_002415.1:m.-173. According to the more accurate transcript annotation (NM_002415.2), the G>C substitution is located at position NM_002415.2:m.-178 and NM_002415.2:c.-270 G>C. (The start codon is located at position chr22:23,894,475.)

DNA was extracted from EDTA-anticoagulated whole blood with Autopure (Qiagen, Hilden, Germany). Genotyping of the SNP rs755622 G>C was performed using an Assays-on-Demand^®^ allelic discrimination on a TaqMan platform according to the manufacturer’s instructions (ThermoFisher Scientific, Waltham, MA, USA). The polymerase chain reaction (PCR) contained 10 ng of genomic DNA, 10 μL TaqMan master mix, and 0.125 μL of 40× assay mix. PCR was performed using 96-well plates on an ABI 9700 thermal cycler (Applied Biosystems, Foster City, CA, USA) (reaction conditions 50 °C for 2 min, 95 °C for 10 min, followed by 40 cycles of 95 °C for 15 s and 60 °C for 1 min). The TaqMan 7700 platform was used to perform an end-plate reading using the allelic discrimination option.

Sample and marker quality control (QC) was performed with PLINK (v1.9; https://www.cog-genomics.org/plink/1.9)(PMID:25722852).

### 2.6. Statistics

Baseline characteristics were analyzed using of Wilcoxon Rank Sum test for continuous variables and Fisher’s exact test for binary variables. Associations between *MIF* polymorphisms and outcome parameters were analyzed using univariate logistic regression and results are presented together with odds ratios and 95% confidence intervals.

Multivariable logistic regression analysis was performed to analyze the influence of relevant baseline variables. Model selection was based on the postoperative complication (dependent variable) and the genotype (independent variable). Further variables were selected on the basis of univariate analyses and significant differences. Model selection was accomplished by a backward elimination or a stepwise procedure.

Serum MIF levels at individual time points were compared using Wilcoxon Rank Sum test according to the *MIF* genotype. Analyses were calculated with SAS 9.4 (SAS Institute Inc., Cary, NC, USA) and SPSS (IBM SPSS, version 21.0, Armonk, NY, USA). All *p*-Values refer to two-sided tests, and *p* < 0.05 was considered statistically significant.

### 2.7. Study Approval

Each patient provided written informed consent prior to inclusion in the study.

## 3. Results

### 3.1. Patients, Baseline Characteristics, and Postoperative Complications

The median age was 68 years and 25.1% were females (Table 1). Postoperative AKI was associated with older age (*p* < 0.001), female gender (*p* = 0.007), the intake of aspirin (*p* = 0.01), lower baseline hemoglobin (<14 g/dL) (*p* < 0.001), peripheral artery disease (*p* = 0.02), hypertension (*p* = 0.01), insulin-dependent diabetes mellitus (IDDM) (*p* = 0.01), and a higher EuroSCORE (*p* < 0.001) (Table 1). Death was associated with older age (*p* = 0.03) and IDDM (*p* = 0.03) (Table 1).

### 3.2. Genotype Frequencies

**Figure 1:**
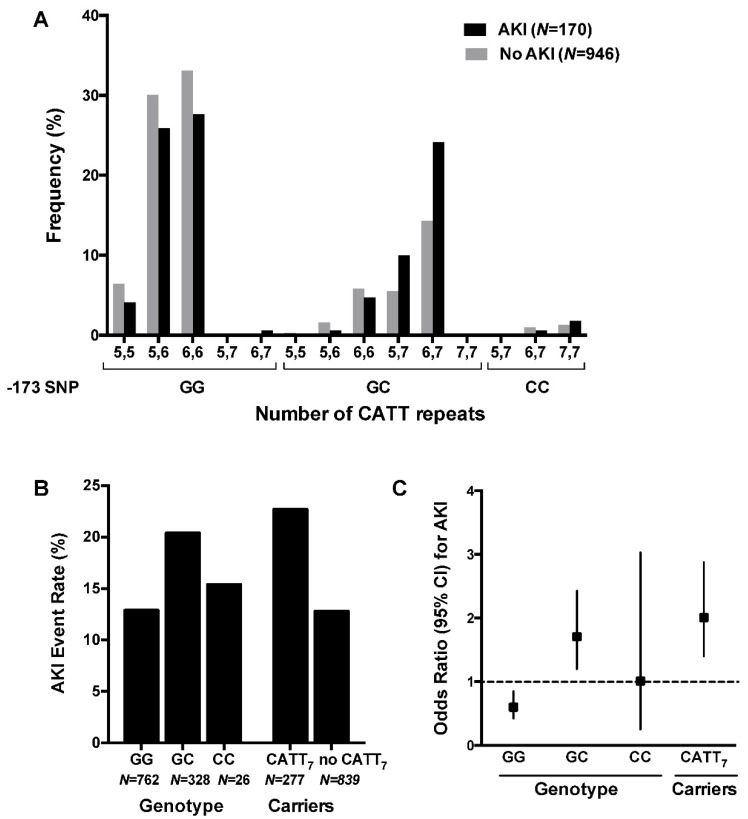
Frequency distribution of the CATT microsatellite repeat (rs3063368) and the G>C SNP (rs755622) in patients with AKI versus patients not affected by AKI after cardiac surgery. (**A**) In patients with AKI, the frequency of the GC genotype and the CATT_7_ allele is higher than in patients without AKI (*Χ^2^* test for trend, *p* = 0.001). (**B**) The AKI event rate was higher in patients with the GC genotype (20%) versus patients with the CC or the GG genotype (13%), and higher in CATT_7_ carriers (23%) compared to non-carriers. (**C**) Patients carrying the GC genotype or the CATT_7_ allele had a significantly increased relative risk of AKI compared to all other patients.

The most common genotypes were CATT_6,6_-GG (32.3%) and CATT_5,6_-GG (30.9%) (Table 2); 24.8% of patients carried at least one CATT_7_ allele; 29.6% of patients were heterozygous and 2.3% of patients were homozygous carriers of the G>C substitution. The allele frequencies in our study cohort were comparable to the frequencies published in the reference database gnomAD (Table 1) [20]. The frequency of the GC genotype and the CATT_7_-repeat allele was higher than in patients with AKI compared to patients without AKI (*Χ^2^* test for trend, *p* = 0.001) (Figure 1).

### 3.3. Association of the Tetranucleotide Repeat CATT_5-7_ (rs3063368) and the G>C Single-Nucleotide Polymorphism (rs755622) with Postoperative Outcome

All patients (*N* = 1116) were examined for the association between the two polymorphisms in the *MIF* promoter and risk of postoperative complications. The overall incidence of AKI after cardiac surgery was 15.2% (*N* = 170) (Table 3). Patients who were either homozygous or heterozygous carriers of the *MIF* CATT_7_ allele had a significantly increased risk of AKI after cardiac surgery when compared to all other patients (22.7% vs. 12.8%, OR 2.01, 95% CI 1.40–2.88, *p* = 0.0001) (Table 4).

Patients carrying the G>C SNP were also at increased risk of AKI (20.4% vs. 13.1%, OR 1.71, 95% CI 1.20–2.43, *p* = 0.0025) (Table 4). The 26 homozygote carriers of the CC genotype did not show an increased risk of AKI when compared to others (*p* = 1.000).

Multiple complications, defined as at least two of the predefined organ dysfunctions, were observed in 12.4% of patients (*N* = 139) (Table 3). The CATT_7_ repeat and the G>C SNP were significantly associated with the occurrence of multiple postoperative complications when compared to all other genotypes (CATT_7_: 17.7% vs. 10.7%, OR 1.79, 95% CI 1.20–2.65, *p* = 0.003; GC: 16.8% vs. 10.7%, OR 1.69, 95% CI 1.15–2.47, *p* = 0.007) (Table 5).

The incidence of death during the first 30 days after surgery was 0.7% (*N* = 8) (Table 3). The mortality of carriers of the *MIF* CATT_7_ allele was significantly higher compared to patients not carrying the CATT_7_ repeat (1.81% vs. 0.36%, *p* = 0.026, OR 5.12, 95% CI 0.99–33.14) (Table 6). Likewise, patients with the *MIF* CATT_6,7_ repeat genotype also had an increased risk of death when compared to all other patients (2.1% vs. 0.4%, OR 4.99, 95% CI 0.92–26.98, *p* = 0.032). Mortality rates in carriers of the GC genotype were increased with borderline significance (1.5% vs. 0.4%, OR 4.05, 95% CI 0.78–26.20, *p* = 0.053).

There was no significant difference in the incidence of AKI or death in heterozygous or homozygous carriers of the C allele or the CATT7 repeat allele (AKI-C-allele: 20.4% vs. 15.4%, OR 1.41 (0.47–4.24), *p* = 0.537; AKI-CATT7: 21.7% vs. 18.8%, OR 1.11 (0.30–4.05), *p* = 0.879; death-C allele: 0% vs. 1.5%, OR 0.90 (0.05–16.75), *p* = 0.526; death-CATT7: 0% vs. 1.9%; OR 0.66 (0.03–12.54), *p* = 0.589). While *MIF* promoter polymorphisms were significantly associated with AKI, multiple complications, and death, no significant association was found with regards to the incidence of postoperative myocardial infarction, atrial fibrillation, stroke, or delirium (Supplemental Tables S2–S5).

### 3.4. MIF Genotypes as a Predictor of AKI in Multivariable Analyses

To assess if the *MIF* genotype improves preoperative risk prediction, a multivariable logistic regression was performed for AKI and death. For risk modeling, all baseline patients’ characteristics (Table 1), including the well-established EuroSCORE, a preoperative risk stratification tool, were considered, and a logistic regression parameter selection procedure was performed. For the prediction of AKI, the variables EuroSCORE, hemoglobin, hypertension, and the presence of the *MIF* CATT_7_ allele were selected. When adjusted for these variables, the *MIF* CATT_7_ allele remained a significant predictor of AKI (OR 2.13, 95% CI, 1.46–3.1) (Table 7). The resulting model had an AUC of 0.71 (95% CI, 0.67–0.76).

**Figure 2:**
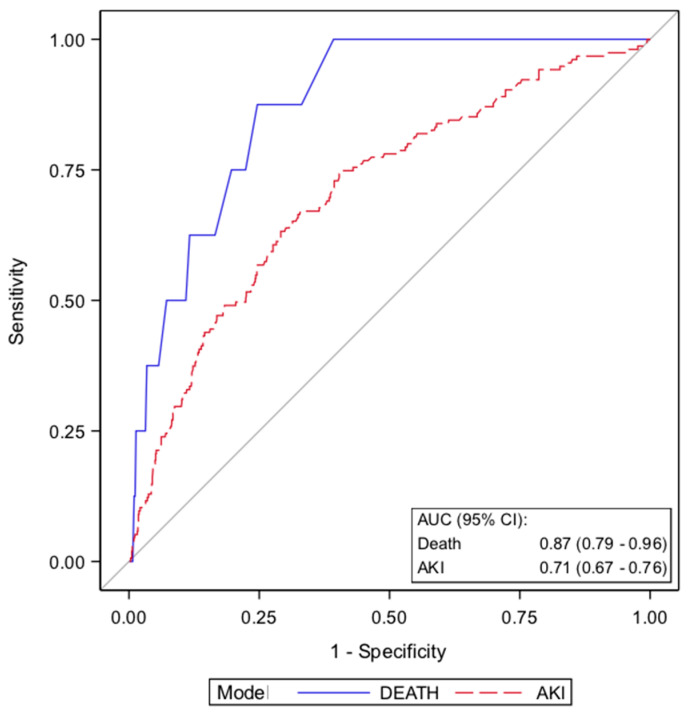
Receiver operating characteristics (ROC) curves for the prediction of AKI (red) and death (blue) after elective cardiac surgery. The grey line indicates the reference values of a diagnostic test that is no better than chance level.

For prediction of death, the statistical variable selection procedure resulted in a model containing the variables EuroSCORE, insulin-dependent diabetes, and the *MIF* CATT_7_ allele. The presence of a *MIF* CATT_7_ repeat allele significantly improved the prediction of postoperative mortality in this model (OR 5.58, 95% CI 1.29–24.04, *p* = 0.021). The resulting model had an AUC (receiver operating statistics—area under the curve) of 0.874 (95% CI, 0.786–0.962) (Table 7, Figure 2). In summary, the *MIF* CATT_7_ allele is a significant predisposing risk factor for AKI and death after cardiac surgery.

The multivariable logistic regression model for AKI includes the variables *MIF* CATT_7_ allele carriers and arterial hypertension as binary variables, and EuroSCORE and hemoglobin levels as continuous variables (according to Table 7). The model for death includes the variables *MIF* CATT_7_ carrier status and insulin-dependent diabetes as binary variables and EuroSCORE as a continuous variable. AUC, area under the curve; CI, confidence interval.

### 3.5. MIF Serum Levels Before Surgery Are Increased in Patients Carrying the CATT_7_ Allele

**Figure 3:**
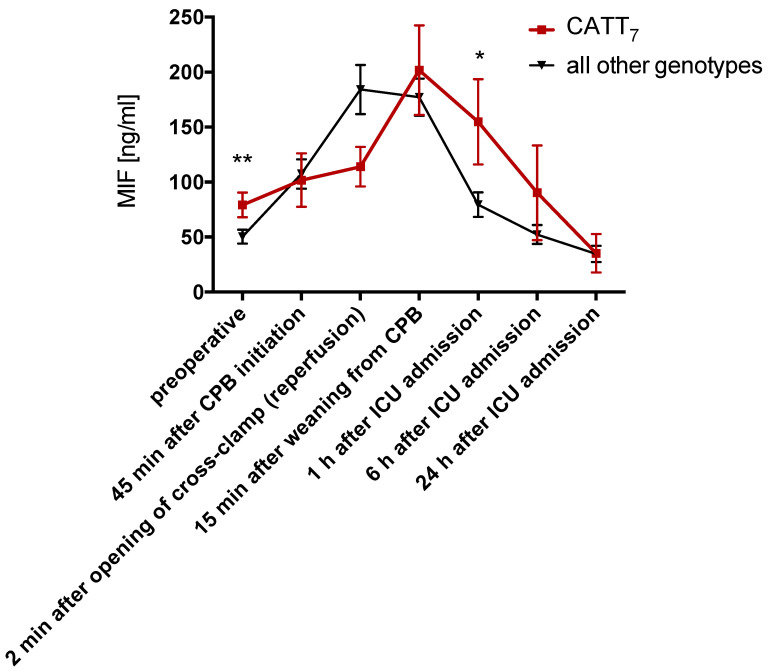
Perioperative kinetics of serum MIF in patients undergoing cardiac surgery. * *P* < 0.05, ** *P* < 0.01.

To assess the association of the *MIF* promoter polymorphisms and the circulating MIF levels in cardiac surgery patients, perioperative kinetics of serum MIF were analyzed in 100 patients in relation to the underlying *MIF* polymorphisms. In patients carrying at least one CATT_7_ allele (CATT_7_), serum MIF was significantly elevated before surgery (79.2 vs. 50.4 ng/mL, *p* = 0.008) and one hour after surgery (154.8 vs. 79.5 ng/mL, *p* = 0.02) (Figure 3). The comparison of all other alleles, genotypes, and individual genotype combinations did not show significant differences between groups.

Serum MIF was quantified with ELISA in 100 patients. Patients heterozygous or homozygous for the *MIF* CATT_7_ allele had significantly increased serum MIF before surgery (preoperative) and significantly lower serum MIF 1 h after surgery. Data are means ± SEM. ** *p* < 0.01, * *p* < 0.05 versus other groups at the corresponding time point (difference between groups) analyzed by Wilcoxon Rank Sum test.

## 4. Discussion

MIF is an inflammatory cytokine that is rapidly released from preformed intracellular pools in response to diverse cellular and systemic stressors, including ischemia-reperfusion, endotoxemia and surgery [21]. Previous studies demonstrated a significant peak of circulating MIF during cardiac surgery, as well as an association between circulating MIF and adverse postoperative outcomes [5,22,23,24]. Two polymorphisms in the *MIF* gene regulatory region, a CATT_5-7_ microsatellite repeat (rs3063368) and a -270 (formerly described as -173) G>C single-nucleotide polymorphism (rs755622), which is in linkage disequilibrium with CATT_7_, have been studied regarding their association with different pathologic conditions (e.g., pulmonary tuberculosis, acute coronary syndrome, carotid artery atherosclerosis, systemic lupus erythematosis, multiple sclerosis) [8,14,25,26,27,28]. However, the impact of the *MIF* gene polymorphisms on postoperative outcomes after cardiac surgery has not been analyzed.

The cardiac surgery patients in the present study displayed similar allele frequencies of the *MIF* CATT_5-7_ microsatellite repeat (rs3063368) and G>C single nucleotide variant (rs755622) as in the general population [20]. Approximately 25% of patients carried at least one longer CATT repeat (CATT_7_) (rs3063368) and 30% carried at least one C allele (rs755622). Demographic characteristics and procedural data revealed no significant differences between genotypes. Heterozygous or homozygous carriers of the CATT_7_ allele had an almost 5-fold increased risk of death after cardiac surgery. Patients carrying either the CATT_7_ or the C allele had an approximately 2-fold increased risk of suffering from AKI or multiple organ dysfunctions. While the risk for AKI was 12.8% in patients not carrying a CATT_7_ allele, it was 22.7% for carriers of the CATT_7_ allele. In addition to the well-established EuroSCORE, the *MIF* CATT_7_ was identified as a significant predictor of death and development of AKI in a multivariable logistic regression model. The reason why we observed no significant association between homozygosity of the CC genotype might be attributed to the very low number of patients (*N* = 26, frequency 2.3%), but should be reevaluated in larger cohorts.

The *MIF* promoter microsatellite was suggested to influence MIF mRNA expression, as assessed by luciferase reporter assays in normal and patient cells [11,14]. The longer CATT_7_ repeat further has been associated with higher serum MIF levels in various patient cohorts, including those with coronary artery disease [10,29,30]. In this study, we found that patients carrying the CATT_7_ allele had higher serum MIF preoperatively and one hour after surgery, but serum MIF did not differ at the other intra- and postoperative timepoints.

In recent literature, MIF has already been associated with AKI in divergent clinical settings of septic shock, liver transplantation, glomerulonephritis, and renal allograft rejection [31,32,33,34].

However, there are indications that MIF can have a protective role against renal tubular injury in experimental models of ischemic AKI [23,35]. In one study, high MIF serum levels during cardiac surgery were associated with a reduced risk of postoperative AKI [23]. This contrasts with our observation of the CATT_7_ and G>C, that are the proposed high expression alleles, being associated with AKI. An important risk factor for postoperative AKI is pre-existing chronic kidney disease (CKD), genetic studies support an association between the presence of at least one *MIF* G>C allele (rs755622) with chronic kidney and cardiovascular disease [36,37]. In a cross-sectional study MIF serum levels were significantly increased in patients with CKD [38]. It is well established that chronic kidney disease (CKD) predisposes to AKI [39]. Therefore, it can be speculated that the association between proposed high expression *MIF* genotypes with AKI is related to CKD that may inflate the risk for postoperative AKI. We suggest that preoperatively, chronically elevated MIF serum levels are associated with detrimental effects and may need to be discriminated from high intraoperative MIF serum levels, which might in fact mediate organ-protective effects during ischemia-reperfusion. This is in line with the notion that acute elevations of MIF serum levels may ameliorate ischemia-reperfusion injury after cardiac surgery, whereas long-term elevations in MIF may aggravate inflammatory pathways in atherosclerosis [40,41]. The observation of MIF correlating with markers of oxidative stress (8-hydroxy-2-deoxyguanosine) and endothelial activation (ICAM-1) in a cohort of CKD patients, supports the thesis that chronically elevated MIF levels might contribute to cardiovascular and associated CKD [38].

Mechanistically, MIF induces intracellular signal cascades via binding to its receptors including the cardioprotective CD74/AMPK kinase axis and the MIF CXC motif chemokine receptors, CXCR2, CXCR4, or CXCR7. Serum MIF may influence the expression of MIF receptors, and there are indications from mouse models that genetic *MIF* deficiency downregulates the expression of the MIF-signalling co-receptor CD44, which is required for signaling responses through CD74 [42,43]. Accordingly, chronically elevated levels of MIF might lead to an altered or injurious response to an acute, perioperative MIF increase, and potentially explain why MIF may be renoprotective in healthy mice but deleterious in multimorbid patients with chronic, underlying inflammation. The results of the present study nevertheless remain observational and cannot explain causative pathophysiology. Further studies investigating the mechanisms by which high expression of the *MIF* genotype may mediate the observed deleterious effects may help in the development of protective strategies for high risk cardiac surgery patients.

We acknowledge several limitations of our study, including the observational design. The event rate for death was very low, and therefore the analysis addressing the association of *MIF* genotypes with mortality should be interpreted cautiously and requires validation in larger cohorts. Although we measured serum MIF in a subcohort of patients and observed a relationship with increased CATT repeat length, this observation has not been consistently observed in prior studies, in part due to limitations of the serum compartment in reflecting systemic or regional tissue *MIF* expression levels [44,45]. Moreover, the study focused on Caucasian subjects and population stratification at the *MIF* locus exists [46]. While we did not study different geographic cohorts, our homogenous study population allows generalisability across predominantly European populations. Finally, the genotyping technique employed does not allow a concise phase analysis, and single haplotypes, e.g., the co-localization of a specific CATT allele (rs3063368) with the G or C allele (rs755622), cannot be explored.

In the same study cohort, one genome-wide association study (GWAS) has been undertaken and did not identify an association of the *MIF* gene polymorphisms with postoperative outcome [47]. Therefore, our findings underscore the necessity of candidate gene studies, including of common structural variants such as microsatellite repeats that are not detectable by SNP-based GWAS platforms [47,48,49]. As technological advances and declining costs in next-generation sequencing technologies pave the way for the broader availability of genomic testing, the implementation of genetic susceptibility data will help to improve risk stratification and to reduce the incidence and sequelae of cardiac surgery [50].

## 5. Conclusions

In the setting of cardiac surgery, we identified the *MIF* promotor polymorphisms CATT_7_ (rs3063368) and -270 (formerly -173) G>C (rs755622), to be predictive of development of postoperative AKI and death. These *MIF* promoter alleles could improve current clinical risk prediction models and thus serve as a helpful decision-making tool for clinicians and patients in the near future.

## Figures and Tables

**Table 1:** Baseline and operative characteristics by AKI and death.

	AKI					Death				
	Yes (*N* = 170)		No (*N* = 946)		*p*-Value	Yes (*N* = 8)		No (*N* = 1108)		*p-*Value
Demographics										
Age, years	72	(66–77)	67	(58–73)	**<0.001**	73	(70–80)	68	(59–73)	**0.03**
Sex (female)	57	(33.5)	223	(23.6)	**0.007**	3	(37.5)	277	(25.0)	0.42
Active smokers	32	(18.8)	198	(20.9)	0.61	1	(12.5)	229	(20.7)	1.00
Medication										
Beta blockers	109	(64.1)	587	(62.1)	0.67	6	(75.0)	690	(62.3)	0.72
ACE inhibitors	91	(53.5)	478	(50.5)	0.51	3	(37.5)	566	(51.1)	0.50
Cholesterol-lowering drug	110	(64.7)	624	(66.0)	0.79	6	(75.0)	728	(65.7)	0.72
Insulin	21	(12.4)	77	(8.1)	0.08	3	(37.5)	95	(8.6)	**0.03**
Aspirin	128	(75.3)	619	(65.4)	**0.01**	4	(50.0)	743	(67.1)	0.45
Clopidogrel	17	(10)	76	(8.0)	0.37	2	(25.0)	91	(8.2)	0.14
Comorbidities										
Hypertension	153	(90.0)	772	(81.6)	**0.01**	6	(75.0)	919	(82.9)	0.63
Ischemic heart disease	132	(77.6)	706	(74.6)	0.39	5	(62.5)	833	(75.2)	0.42
Previous MI						1	(12.5)	315	(28.4)	0.45
Congestive heart disease	43	(25.3)	193	(20.4)	0.15	2	(25.0)	234	(21.1)	0.68
PAD	20	(11.8)	62	(6.6)	**0.02**	2	(25.0)	80	(7.2)	0.68
COPD	15	(8.8)	80	(8.5)	0.88	0	0	95	(8.6)	1.00
Chronic kidney disease	24	(14.1)	107	(11.3)	0.30	1	(12.5)	130	(11.7)	1.00
IDDM	56	(32.9)	219	(23.2)	**0.01**	4	(50.0)	271	(24.5)	0.11
Laboratory, at baseline										
Serum creatinine mg/dL	0.92	(0.8–1.1)	0.91	(0.8–1.1)	0.66	0.99	(0.73–1.14)	0.91	(0.80–1.07)	0.82
Hemoglobin, g/dL	13.6	(12.5–14.6)	14.2	(13.4–14.9)	**<0.001**	13.6	(12.1–14.4)	14.1	(13.2–14.9)	0.31
EuroSCORE										
	5	(3–7)	4	(2–6)	**<0.001**	6	(5–8)	4	(2–6)	**0.03**
Type of surgery										
CABG (alone)	64	(37.6)	434	(45.9)	0.26	3	(37.5)	495	(44.7)	0.25
Aortic valve *	37	(12.8)	190	(20.1)		2	(12.5)	225	(20.3)	
Mitral valve *	6	(3.5)	32	(3.4)		0	(37.5)	38	(3.4)	
Aorta ascendens *	3	(1.8)	30	(3.2)		0	(25.0)	33	(3.0)	
Combined procedures	57	(33.5)	245	(25.9)		2	25.0	300	(27.1)	
Other type of surgery	3	(1.8)	15	(1.6)		1	12.5	17	(1.5)	

Data are expressed as the median and interquartile range (Q1–Q3) or absolute numbers and (percentage). The association of baseline characteristics with AKI and death was analyzed by Wilcoxon rank sum or Fisher’s exact test. ACE, angiotensin converting enzyme; CABG, coronary artery bypass graft; COPD, chronic obstructive pulmonary disease; IDDM, insulin-dependent diabetes mellitus; MI, myocardial infarction; PAD, peripheral artery disease, *, replacement or reconstruction (alone). Bold fonts indicate *p*-values < 0.05.

**Table 2:** Frequencies of the *MIF* CATT_5–7_ repeat allele (rs3063368) and the G>C single-nucleotide polymorphism (rs755622) in 1116 patients undergoing cardiac surgery.

**CATT_5–7_ Repeat Allele Carrier Frequencies (rs3063368)**			
	***N Carriers***	**%**	**Allele Frequency (Europe) % ^1^**
CATT_5_	488	43.7	- *
CATT_6_	957	85.8	84.3
CATT_7_	277	24.8	24.9
**G>C SNP Genotype Frequencies (rs755622)**			
	***N* Genotypes**	**%**	**Allele Frequency (Europe) % ^1^**
GG (homozygous)	762	68.3	65.2
GC (heterozygous)	328	29.4	31.7
CC (homozygous)	26	2.3	3.1
**Individual Genotype Combination Frequencies (rs3063368 & rs755622)**			
	***N***	**%**	
CATT_5,5_-GG	68	6.1	
CATT _5,6_-GG	329	29.5	
CATT _6,6_-GG	360	32.3	
CATT _5,7_-GG	2	0.2	
CATT _6,7_-GG	3	0.3	
CATT _5,5_-CG	3	0.3	
CATT _5,6_-CG	16	1.4	
CATT _6,6_-CG	63	5.6	
CATT _5,7_-CG	69	6.2	
CATT _6,7_-CG	176	15.8	
CATT _7,7_-CG	1	0.1	
CATT _5,7_-CC	1	0.1	
CATT _6,7_-CC	10	0.9	
CATT _7,7_-CC	15	1.3	
All	1116	100.00	

The most frequent genotypes observed were CATT _5,6_-GG (29.5%), CATT _6,6_-GG (32.3%), and CATT _6,7_-CG (15.8%). The frequency of the polymorphisms was comparable to the frequency in the general reference population (Europe) ^1^; SNP, Single nucleotide polymorphism; CATT_7_, patients carrying at least one CATT_7_ allele. Data presented as absolute numbers and percentage. ^1^ calculated from reference gnomAD Database (including >7500 genomes from unrelated non-Finnish European individuals sequenced as part of various population genetic studies) [20]. * As CATT_5_ is the wildtype allele, there is no information regarding CATT_5_ in the gnomAD Database.

**Table 3:** Absolute and relative frequency of postoperative complications. * Patients affected by at least two of the predefined organ dysfunctions.

		Patients (*N* = 1116)	
		*N*	%
Death		8	0.7
Myocardial infarction (MI)		93	8.3
Stroke		24	2.2
Delirium		144	12.9
Acute kidney injury (AKI)		170	15.2
	Stage 1	108	9.7
	Stage 2	38	3.4
	Stage 3	24	2.1
Atrial Fibrillation		245	21.9
Multiple Complications (≥) *		139	12.4

**Table 4:** Association of the *MIF* promoter polymorphisms with AKI.

*MIF* Polymorphism		AKI (*N* = 170)						*p*-Value
		Patients Carrying This Allele/Genotype		Patients NOT Carrying this Allele/Genotype				
		*N*	Incidence, %	*N*	Incidence, %	OR	(95% CI)	
**CATT Repeat** **Allele Carriers** **(rs3063368)**								
	CATT_5_	69	14.1	101	16.1	0.86	(0.61–1.21)	0.401
	CATT _6_	143	14.9	27	17.0	0.86	(0.54–1.40)	0.551
	CATT _7_	**63**	**22.7**	**107**	**12.8**	**2.01**	**(1.40–2.88)**	**0.0001**
**Genotypes**								
G>C (rs755622)								
	**GG**	**99**	**12.9**	**71**	**20.1**	**0.60**	**(0.42–0.85)**	**0.0031**
	**GC**	**67**	**20.4**	**103**	**13.1**	**1.71**	**(1.20–2.43)**	**0.0025**
	CC	4	15.4	166	15.2	1.01	(0.25–3.03)	1.000
CATT repeat (rs3063368)								
	CATT _5,5_	7	9.9	163	15.6	0.59	(0.22–1.32)	0.233
	CATT _5,6_	45	13.0	125	16.2	0.78	(0.52–1.13)	0.178
	CATT _5,7_	17	23.6	153	14.7	1.80	(0.95–3.25)	0.060
	CATT _6,6_	55	13.0	115	16.6	0.75	(0.52–1.07)	0.122
	**CATT _6,7_**	**43**	**22.8**	**127**	**13.7**	**1.86**	**(1.23–2.77)**	**0.003**
	CATT _7,7_	3	18.8	167	15.2	1.29	(0.23–4.76)	0.723
**Individual** genotype combinations *								
	CATT _5,5_-GG (6.1%)	7	10.3	163	15.6	0.62	(0.24–1.40)	0.297
	CATT _5,6_-GG (29.5%)	44	13.4	126	16.0	0.81	(0.55–1.19)	0.275
	CATT _6,6_-GG (32.3%)	47	13.1	123	16,3	0.77	(0.53–1.12)	0.182
	CATT _6,6_-CG (5.6%)	8	12.7	162	15.4	0.80	(0.32–1.73)	0.718
	**CATT _5,7_-CG (6.2%)**	**17**	**24.6**	**153**	**14.6**	**1.91**	**(1.01–3.46)**	**0.036**
	**CATT _6,7_-CG (15.8%)**	**41**	**23.3**	**129**	**13.7**	**1.91**	**(1.25–2.87)**	**0.002**

The incidence of AKI was higher among patients carrying the CATT_7_ allele, in patients with the GC or CATT _6,7_ genotype, and in patients with the genotype combinations CATT _5,7_-CG and CATT _6,7_-CG. AKI, acute kidney injury; CI, confidence interval; OR, odds ratio; SNP, Single nucleotide polymorphism; CATT_7_, patients carrying at least one CATT_7_ allele. * genotype combinations with a frequency of >5%. Data presented as absolute numbers and percentage. *p*-value calculated by Fisher exact test; bold fonts indicate *p*-values < 0.05.

**Table 5:** Association of the *MIF* promoter polymorphisms with multiple complications *.

*MIF* Polymorphism		Multiple Complications * (*N* = 139)						*p*-Value
		Patients Carrying this Allele/Genotype		Patients NOT Carrying this Allele/Genotype				
		*N*	Incidence, %	*N*	Incidence, %	OR	(95% CI)	
**-CATT repeat** **allele carriers** **(rs3063368)**								
	CATT _5_	50	10.3	89	14.2	0.69	(0.47–1.01)	0.055
	CATT _6_	120	12.5	19	12.0	1.06	(0.62–1.88)	0.898
	CATT _7_	**49**	**17.7**	**90**	**10.7**	**1.79**	**(1.20–2.65)**	**0.003**
**Genotypes**								
G>C (rs755622)								
	**GG**	**80**	**10.1**	**59**	**16.7**	**0.59**	**(0.40–0.86)**	**0.005**
	**GC**	**55**	**16.8**	**84**	**10.7**	**1.69**	**(1.15–2.47)**	**0.007**
	CC	4	15.4	135	12.4	1.29	(0.32–3.87)	0.554
CATT repeat (rs3063368)								
	CATT _5,5_	4	5.6	135	12.9	0.40	(0.10–1.11)	0.092
	CATT _5,6_	33	9.6	106	13.8	0.66	(0.42–1.02)	0.050
	CATT _5,7_	13	18.1	126	12.1	1.61	(0.78–3.06)	0.140
	CATT _6,6_	53	12.5	86	12.4	1.01	(0.69–1.48)	1.000
	**CATT _6,7_**	**34**	**18.0**	**105**	**11.3**	**1.72**	**(1.09–2.66)**	**0.015**
	CATT _7,7_	2	12.5	137	12.5	1.00	(0.11–4.45)	1.000
**Individual** **genotype combinations ^†^**								
	CATT _5,5_-GG (6.1%)	4	5.9	135	12.9	0.42	(0.11–1.16)	0.126
	CATT _5,6_-GG (29.5%)	32	9.7	107	13.6	0.68	(0.44–1.05)	0.091
	CATT _6,6_-GG (32.3%)	44	12.2	95	12.6	0.97	(0.65–1.44)	0.923
	CATT _6,6_-CG (5.6%)	9	14.3	130	12.4	1.18	(0.50–2.49)	0.693
	**CATT _5,7_-CG (6.2%)**	13	18.4	126	12.0	1.70	(0.83–3.25)	0.127
	**CATT _6,7_-CG (15.8%)**	**32**	**18.2**	**107**	**11.4**	**1.73**	**(1.08–2.70)**	**0.018**

The incidence of multiple complications was higher among patients carrying the CATT_7_ allele, in patients with the GC or CATT _6,7_ genotype, and in patients with the genotype combinations CATT _5,7_-CG and CATT _6,7_-CG. CI, confidence interval; OR, odds ratio; SNP, Single nucleotide polymorphism; CATT_7x_, patients carrying at least one CATT_7_ allele. ^†^ genotype combinations with a frequency of > 5%. * Patients affected by at least two of the predefined organ dysfunctions. ^†^ defined as at least two of the predefined organ dysfunctions. Data presented as absolute numbers and percentage. *p*-value calculated by Fisher’s exact test; bold fonts indicate *p*-values < 0.05.

**Table 6:** Association of the *MIF* promoter polymorphisms with death.

*MIF* Polymorphism		Death (*N* = 8)						*p*-Value
		Patients Carrying This Allele/Genotype		Patients NOT Carrying This Allele/Genotype				
		*N*	Incidence, %	*N*	Incidence, %	OR	(95% CI)	
**CATT repeat** **allele carriers** **(rs3063368)**								
	CATT _5_	2	0.4	6	1.0	0.43	(0.04–2.40)	0.478
	CATT _6_	7	0.7	1	0.6	1.16	(0.15–52.80)	1.000
	**CATT _7_**	**5**	**1.8**	**3**	**0.4**	**5.12**	**(0.99–33.14)**	**0.026**
**Genotypes**								
G>C (rs755622)								
	GG	3	0.4	5	1.4	0.28	(0.04–1.43)	0.118
	GC	5	1.5	3	0.4	4.05	(0.78–26.20)	0.053
	CC	0	0	8	0.7	0.00	(0.00–19.77)	1.000
CATT repeat (rs3063368)								
	CATT _5,5_	0	0	8	0.8	0.00	(0.00–6.75)	1.000
	CATT _5,6_	1	0.3	7	0.9	0.32	(0.01–2.49)	0.447
	CATT _5,7_	1	1.4	7	0.7	2.09	(0.05–16.59)	0.415
	CATT _6,6_	2	0.5	6	0.9	0.54	(0.05–3.06)	0.717
	**CATT _6,7_**	**4**	**2.1**	**4**	**0.4**	**4.99**	**(0.92–26.98)**	**0.032**
	CATT _7,7_	0	0	8	0.7	0.00	(0.00–33.33)	1.000
**Individual** genotype combinations *								
	CATT _5,5_-GG (6.1%)	0	0	8	0.8	0.00	(0.00–7.07)	1.000
	CATT _5,6_-GG (29.5%)	1	0.3	7	0.9	0.34	(0.01–2.66)	0.449
	CATT _6,6_-GG (32.3%)	2	0.6	6	0.8	0.70	(0.07–3.93)	1.000
	CATT _6,6_-CG (5.6%)	0	0	8	0.8	0.00	(0.00–7.68)	1.000
	CATT _5,7_-CG (6.2%)	1	1.5	7	0.7	2.18	(0.05–17.39)	0.401
	**CATT _6,7_-CG (15.8%)**	**4**	**2.3**	**4**	**0.4**	**5.44**	**(1.00–29.44)**	**0.025**

The incidence of death was higher among patients carrying the CATT_7_ allele, in patients with the CATT _6,7_ genotype, and in patients with the genotype combination CATT _6,7_-CG.; OR, odds ratio; SNP, Single nucleotide polymorphism; CATT_7_, patients carrying at least one CATT_7_ allele. * genotypes with a frequency of > 5%. Data presented as absolute numbers and percentage. *p*-value calculated by Fisher’s exact test; bold fonts indicate *p*-values < 0.05.

**Table 7:** Predictors of Acute Kidney Injury (AKI) and death using multivariable logistic regression.

Variable	ß	OR	(95% CI)	*p*-Value
AKI (*N* = 170) *	−1.066			
CATT_7_ carrier	0.755	2.13	(1.46–3.09)	**<0.001**
EuroSCORE	0.202	1.22	(1.38–1.32)	**<0.001**
Hemoglobin	−0.183	0.83	(0.74–0.94)	**0.004**
Hypertension	0.701	2.03	(1.11–3.72)	**0.022**
**Death (*N* = 8) ^†^**	−6.709			
CATT_7_ carrier	1.719	5.58	(1.29–24.04)	**0.021**
EuroSCORE	0.342	1.41	(1.05–1.88)	**0.021**
IDDM	1.923	6.84	(1.55–30.26)	**0.011**

In addition to the well-established EuroSCORE and other baseline characteristics, the *MIF* CATT_7_ was identified as a significant predictor of AKI and death. AKI, acute kidney injury; OR, odds ratio; CI, confidence interval. IDDM, insulin-dependent diabetes mellitus. Bold fonts indicate *p*-values < 0.05. * Area under the curve (AUC), 0.71; 95% CI, 0.67 - 0.76; Hosmer und Lemeshow Goodness-of-Fit Test, 0.8432. **^†^** Area under the curve (AUC), 0.87; 95% CI, 0.79–0.96; Hosmer und Lemeshow Goodness-of-Fit Test, 0.9702.

## Supplementary Materials

The following are available online at https://www.mdpi.com/2077-0383/9/9/2936/s1. Supplemental Figure S1. Flowchart of the patients screened and included in the study. Supplemental Figure S2. SNP rs5844572 region (CATT_n_ tetranucleotide repeat) and rs755622 (G>C) with UCSC genome browser (Geb. 2009; GRCh37/hg19 Assembly). Supplemental Table S1. Primer sequences used for genotyping of the tetranucleotide repeat polymorphism CATT_n_ (rs3063368). Supplemental Table S2. Association of *MIF* promoter polymorphisms with Myocardial Infarction. Supplemental Table S3. Association of *MIF* promoter polymorphisms with Stroke. Supplemental Table S4. Association of *MIF* promoter polymorphisms with delir. Supplemental Table S5. Association of MIF regulatory polymorphisms with atrial fibrillation.
