# Supplementary material for: The Macrophage Migration Inhibitory Factor (MIF) Promoter Polymorphisms (rs3063368, rs755622) Predict Acute Kidney Injury and Death after Cardiac Surgery
Source: J Clin Med. 2020 Sep 11;9(9):2936. doi: 10.3390/jcm9092936 (PMC7565645; doi:10.3390/jcm9092936)
Supplement: Supplementary file 1 [file jcm-09-02936-s001.pdf]

SUPPLEMENATAL MATERIAL

Supplemental Figure 1. Flowchart of the patients screened and included in the study.

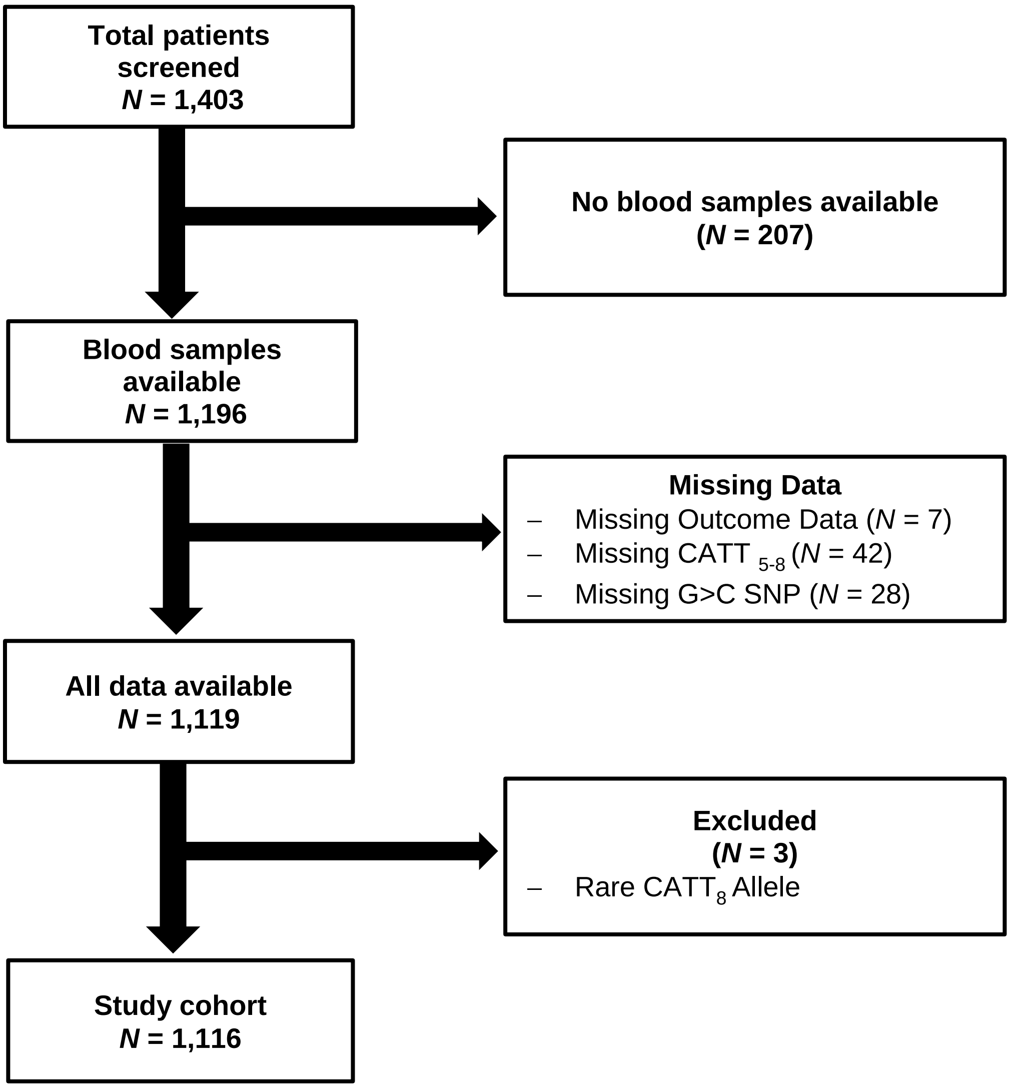

Supplemental Figure 2. SNP rs5844572 region ($CATT_n$ tetranucleotide repeat) and rs755622 (G>C) with UCSC genome browser (Geb. 2009; GRCh37/hg19 Assembly)

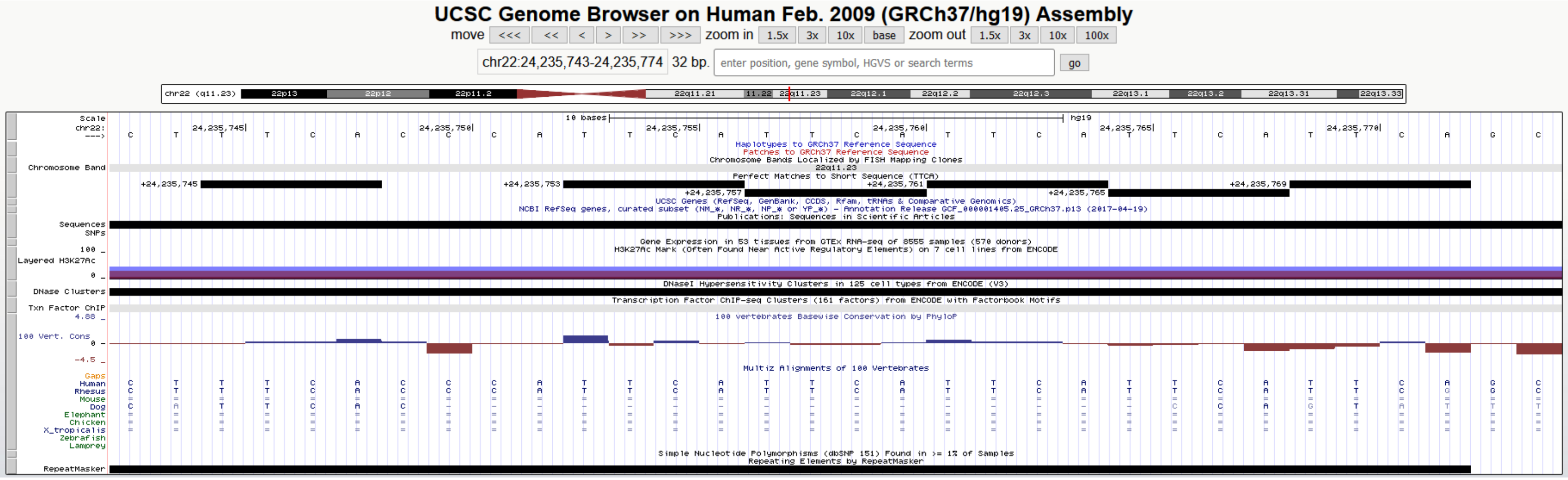

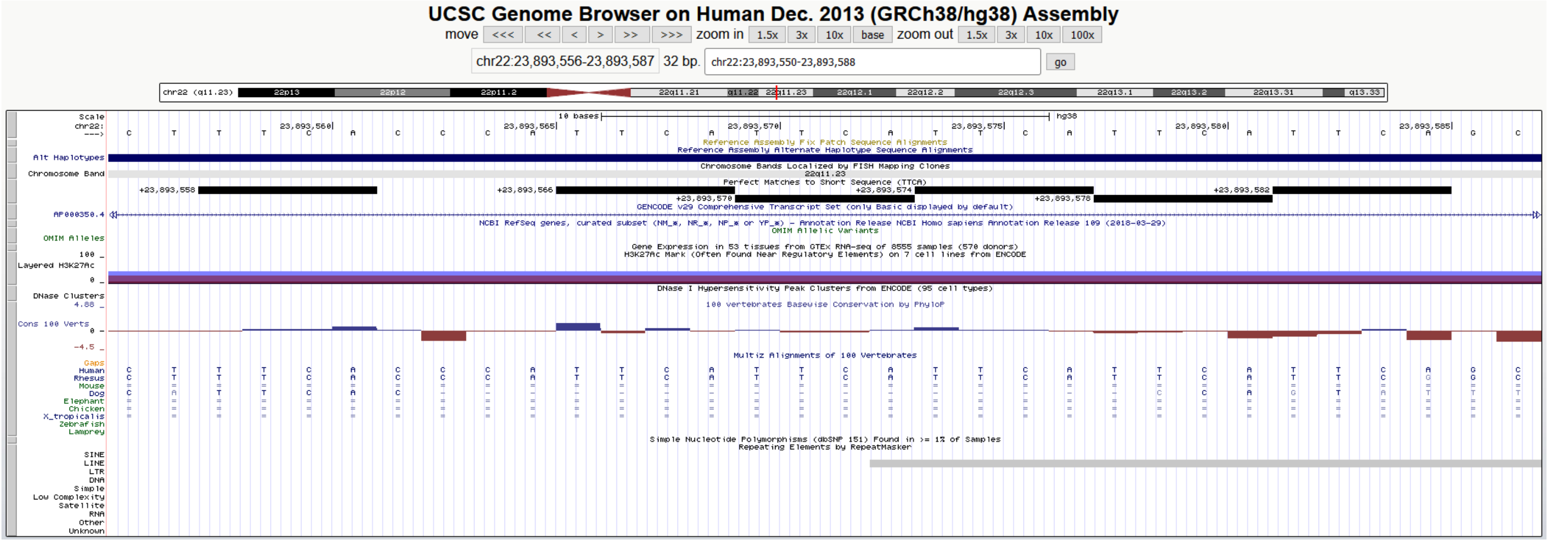

Supplemental Table 1. Primer sequences used for genotyping of the tetranucleotide repeat polymorphism CATT$_n$ (rs3063368)

	Forward primer	Backward primer	Annealing temperature
**Set 1**	ggtcgctatgaacgcacag	cttaaccccagccttccagt	69 °C
**Set 2**	ggtaccctaggcagccaatc	cacggaaagtcagagcttgg	70 °C
**Set 3**	gtatcgggatccctgccc	tgctcgcactttgaagatgg	69 °C
**Set 4**	gctacctcccacacctgag	tctcccagccccagatct	65 °C

MIF Polymorphism	Myocardial infarction (*N* = 93)						*P*-value
	Patients carrying this allele/genotype		Patients NOT carrying this allele/genotype				
	N	Prevalence, %	*N*	Prevalence, %	OR	(95% CI)	
**CATT repeat allele carriers (rs3063368)**							
CATT $_5$	40	8.20	53	8.44	0.97	(0.61-1.52)	0.9134
CATT $_6$	80	8.36	13	8.18	1.02	(0.55-2.06)	1.000
CATT $_7$	27	9.75	66	7.87	1.26	(0.76-2.06)	0.3184
**Genotypes**							
G>C (rs755622)							
**GG**	61	8.01	32	9.04	0.88	(0.55-1.42)	0.562
**GC**	30	9.15	63	7.99	1.16	(0.71-1.86)	0.553
CC	2	7.69	91	8.35	0.91	(0.10-3.78)	1.000
CATT repeat (rs3063368)							
CATT $_{5,5}$	5	7.04	88	8.42	0.82	(0.25-2.10)	0.827
CATT $_{5,6}$	27	7.83	66	8.56	0.91	(0.55-1.47)	0.726
CATT $_{5,7}$	8	11.1	85	8.14	1.41	(0.56-3.08)	0.376
CATT $_{6,6}$	34	8.04	59	8.51	0.94	(0.59-1.49)	0.824
CATT $_{6,7}$	19	10.05	74	7.98	1.29	(0.71-2.22)	0.385
CATT $_{7,7}$	0	0.00	93	8.45	0.00	(0.00-2.27)	0.388
**Individual genotype combinations** #							
CATT $_{5,5}$-GG (6.1%)	5	7.35	88	8.40	0.87	(0.26-2.21)	1.000
CATT $_{5,6}$-GG (29.5%)	25	7.60	68	8.64	0.87	(0.52-1.43)	0.635
CATT $_{6,6}$-GG (32.3%)	31	8.61	62	8.20	1.05	(0.65-1.69)	0.817
CATT $_{6,6}$-CG (5.6%)	3	4.76	90	8.55	0.54	(0.11-1.69)	0.477
CATT $_{5,7}$-CG (6.2%)	8	11.59	85	8.12	1.48	(0.59-3.25)	0.3637
CATT $_{6,7}$-CG (15.8%)	17	9.66	76	8.09	1.22	(0.66-2.14)	0.460

Supplemental Table 2. Association of *MIF* promoter polymorphisms with Myocardial Infarction. Association between two of the polymorphisms in the *MIF* gene and risk of postoperative myocardial infarction. CI, confidence interval; OR, odds ratio; SNP, Single nucleotide polymorphism; CATT$_{7x}$, patients carrying at least one CATT$_7$ allele. # genotypes with a frequency of > 5%. Data presented as absolute numbers and percentage. *P* value calculated by Fisher exact test; bold fonts indicate *P*-values < 0.05.

MIF Polymorphism	Stroke (*N* = 24)						
	Patients carrying this allele/genotype		Patients NOT carrying this allele/genotype				*P*-value
	N	Prevalence, %	*N*	Prevalence, %	OR	(95% CI)	
**CATT repeat allele carriers (rs3063368)**							
$CATT_5$	7	1.43	17	2.71	0.52	(0.18-1.34)	0.211
$CATT_6$	22	2.30	2	1.26	1.85	(0.45-16.35)	0.561
$CATT_7$	6	2.17	18	2.15	1.01	(0.32-2.69)	1.000
**Genotypes**							
G>C (rs755622)							
GG	16	2.10	8	2.26	0.93	(0.37-2.53)	0.828
GC	8	2.44	16	2.03	1.21	(0.44-3.02)	0.655
CC	0	0.00	24	2.20	0.00	(0.00-5.76)	1.000
CATT repeat (rs3063368)							
$CATT_{5,5}$	1	1.41	23	2.20	0.63	(0.02-4.03)	1.000
$CATT_{5,6}$	5	1.45	19	2.46	0.58	(0.17-1.63)	0.373
$CATT_{5,7}$	1	1.39	23	2.20	0.63	(0.01-3.97)	1.000
$CATT_{6,6}$	12	2.84	12	1.73	1.66	(0.67-4.07)	0.287
$CATT_{6,7}$	5	2.65	19	2.05	1.30	(0.37-3.66)	0.583
$CATT_{7,7}$	0	0.00	24	2.18	0.00	(0.00-9.77)	1.000
**Individual genotype combinations** #							
$CATT_{5,5}$-GG (6.1%)	1	1.47	23	2.19	0.67	(0.02-4.23)	1.000
$CATT_{5,6}$-GG (29.5%)	5	1.52	19	2.41	0.62	(0.18-1.75)	0.497
$CATT_{6,6}$-GG (32.3%)	10	2.78	14	1.85	1.51	(0.60-3.71)	0.377
$CATT_{6,6}$-CG (5.6%)	2	3.17	22	2.09	1.54	(0.17-6.49)	0.642
$CATT_{5,7}$-CG (6.2%)	1	1.45	23	2.20	0.65	(0.02-4.16)	1.000
$CATT_{6,7}$-CG (15.8%)	5	2.84	19	2.02	1.42	(0.41-3.99)	0.568

Supplemental Table 3. Association of *MIF* promoter polymorphisms with Stroke

Association between two of the polymorphisms in the *MIF* gene and risk of postoperative stroke. CI, confidence interval; OR, odds ratio; SNP, Single nucleotide polymorphism; $CATT_{7x}$, patients carrying at least one $CATT_7$ allele. # genotypes with a frequency of > 5%. Data presented as absolute numbers and percentage. *P* value calculated by Fisher exact test; bold fonts indicate *P*-values < 0.05.

MIF Polymorphism	Delir (*N* = 144)						
	Patients carrying this allele/genotype		Patients NOT carrying this allele/genotype				*P*-value
	N	Prevalence, %	*N*	Prevalence, %	OR	(95% CI)	
**CATT repeat allele carriers (rs3063368)**							
CATT $_5$	61	12.50	83	13.22	0.94	(0.65-1.36)	0.787
CATT $_6$	119	12.43	25	15.72	0.76	(0.47-1.27)	0.251
CATT $_7$	44	15.88	100	11.92	1.40	(0.93-2.08)	0.098
**Genotypes**							
G>C (rs755622)							
GG	92	12.07	52	14.69	0.80	(0.55-1.18)	0.250
GC	47	14.33	97	12.31	1.19	(0.80-1.76)	0.378
CC	5	19.23	139	12.75	1.63	(0.47-4.53)	0.367
CATT repeat (rs3063368)							
CATT $_{5,5}$	12	16.90	132	12.63	1.41	(0.67-2.73)	0.277
CATT $_{5,6}$	38	11.01	106	13.75	0.78	(0.51-1.17)	0.246
CATT $_{5,7}$	11	15.28	133	12.74	1.24	(0.57-2.45)	0.584
CATT $_{6,6}$	50	11.82	94	13.56	0.85	(0.58-1.25)	0.409
CATT $_{6,7}$	31	16.40	113	12.19	1.41	(0.89-2.21)	0.122
CATT $_{7,7}$	2	12.5	142	12.91	0.96	(0.11-4.27)	1.00
**Individual genotype combinations** #							
CATT $_{5,5}$-GG (6.1%)	12	17.65	132	12.60	1.49	(0.71-2.90)	0.260
CATT $_{5,6}$-GG (29.5%)	36	10.94	108	13.72	0.77	(0.50-1.17)	0.240
CATT $_{6,6}$-GG (32.3%)	43	11.94	101	13.36	0.88	(0.59-1.30)	0.567
CATT $_{6,6}$-CG (5.6%)	7	11.11	137	13.01	0.84	(0.31-1.89)	0.847
CATT $_{5,7}$-CG (6.2%)	11	15.94	133	12.70	1.30	(0.60-2.59)	0.457
CATT $_{6,7}$-CG (15.8%)	27	15.34	117	12.45	1.27	(0.78-2.03)	0.323

Supplemental Table 4. Association of *MIF* promoter polymorphisms with delir Association between two of the polymorphisms in the *MIF* gene and risk of postoperative delir. CI, confidence interval; OR, odds ratio; SNP, Single nucleotide polymorphism; CATT$_{7x}$, patients carrying at least one CATT$_7$ allele. # genotypes with a frequency of > 5%. Data presented as absolute numbers and percentage. *P* value calculated by Fisher exact test; bold fonts indicate *P*-values < 0.05.

MIF Polymorphism	Atrial Fibrillation (*N* = 245)						
	Patients carrying this allele/genotype		Patients NOT carrying this allele/genotype				*P*-value
	N	Prevalence, %	*N*	Prevalence, %	OR	(95% CI)	
**CATT repeat allele carriers (rs3063368)**							
CATT $_5$	104	21.31	139	22.13	0.95	(0.71-1.28)	0.770
CATT $_6$	209	21.84	34	21.38	1.03	(0.67-1.60)	1.000
CATT $_7$	56	20.22	187	22.29	0.88	(0.62-1.25)	0.502
**Genotypes**	**Genotypes**						
G>C (rs755622)	G>C (rs755622)						
GG	170	22.31	73	20.62	1.11	(0.80-1.53)	0.586
GC	69	21.04	174	22.08	0.94	(0.68-1.30)	0.750
CC	4	15.38	239	21.93	0.65	(0.16-1.93)	0.630
CATT repeat (rs3063368)	CATT repeat (rs3063368)						
CATT $_{5,5}$	14	19.72	229	21.91	0.88	(0.44-1.63)	0.767
CATT $_{5,6}$	73	21.16	170	22.05	0.95	(0.69-1.30)	0.754
CATT $_{5,7}$	17	23.61	226	21.65	1.12	(0.60-2.00)	0.660
CATT $_{6,6}$	100	23.64	143	20.63	1.19	(0.88-1.61)	0.122
CATT $_{6,7}$	36	19.05	207	22.33	0.82	(0.54-1.23)	0.335
CATT $_{7,7}$	3	18.75	240	21.82	0.83	(0-15-3.04)	1.000
**Individual genotype combinations** #	**Individual genotype combinations** #						
CATT $_{5,5}$-GG (6.1%)	13	19.12	230	21.95	0.84	(0.41-1.59)	0.652
CATT $_{5,6}$-GG (29.5%)	72	21.88	171	21.73	1.01	(0.73-1.39)	1.000
CATT $_{6,6}$-GG (32.3%)	84	23.33	159	21.03	1.14	(0.83-1.56)	0.394
CATT $_{6,6}$-CG (5.6%)	16	25.40	227	21.56	1.24	(0.64-2.27)	0.529
CATT $_{5,7}$-CG (6.2%)	16	23.19	227	21.68	1.09	(0.57-1.98)	0.764
CATT $_{6,7}$-CG (15.8%)	35	19.89	208	22.13	0.87	(0.57-1.32)	0.551

Supplemental Table 5. Association of MIF regulatory polymorphisms with atrial fibrillation. Association between two of the polymorphisms in the *MIF* gene and risk of postoperative atrial fibrillation. CI, confidence interval; OR, odds ratio; SNP, Single nucleotide polymorphism; CATT$_{7x}$, patients carrying at least one CATT$_7$ allele. # genotypes with a frequency of > 5%. Data presented as absolute numbers and percentage. *P* value calculated by Fisher exact test; bold fonts indicate *P*-values < 0.05.

© 2020 by the authors. Licensee MDPI, Basel, Switzerland. This article is an open access article distributed under the terms and conditions of the Creative Commons Attribution (CC BY) license (http://creativecommons.org/licenses/by/4.0/).
